# Periurban *Trypanosoma cruzi*–infected *Triatoma infestans*, Arequipa, Peru

**DOI:** 10.3201/eid1209.051662

**Published:** 2006-09

**Authors:** Michael Zachary Levy, Natalie M. Bowman, Vivian Kawai, Lance A. Waller, Juan Geny Cornejo del Carpio, Eleazar Cordova Benzaquen, Robert H. Gilman, Caryn Bern

**Affiliations:** * Centers for Disease Control and Prevention, Atlanta, Georgia, USA;; † Emory University, Atlanta, Georgia, USA;; ‡ Asociación Benéfica Proyectos en Informática, Salud, Medicina y Agricultura, Lima, Peru;; § Dirección Regional del Ministerio de Salud, Arequipa, Peru;; ¶ Universidad Nacional San Agustín, Arequipa, Peru;; # Johns Hopkins University, Baltimore, Maryland, USA

**Keywords:** Chagas Disease, Triatoma infestans, Trypanosoma cruzi, Urbanization, Peru

## Abstract

In Arequipa, Peru, vectorborne transmission of Chagas disease by *Triatoma infestans* has become an urban problem. We conducted an entomologic survey in a periurban community of Arequipa to identify risk factors for triatomine infestation and determinants of vector population densities. Of 374 households surveyed, triatomines were collected from 194 (52%), and *Trypanosoma cruzi*–carrying triatomines were collected from 72 (19.3%). Guinea pig pens were more likely than other animal enclosures to be infested and harbored 2.38× as many triatomines. Stacked brick and adobe enclosures were more likely to have triatomines, while wire mesh enclosures were protected against infestation. In human dwellings, only fully stuccoed rooms were protected against infestation. Spatially, households with triatomines were scattered, while households with *T. cruzi*–infected triatomines were clustered. Keeping small animals in wire mesh cages could facilitate control of *T. infestans* in this densely populated urban environment.

## Abstract 2

Simple interventions may facilitate vector control and prevent periurban transmission of Chagas disease.

Chagas disease, caused by the protozoan parasite *Trypanosoma cruzi*, causes more deaths in the Americas than any other parasitic disease (*1*). *T. cruzi* is carried in the gut of bloodsucking triatomine insects (Hemiptera, Reduviidae), and the parasite is usually transmitted to humans when the vector's feces enter the host through the insect bite or mucous membranes (*2*). *Triatoma infestans* is the principal vector of *T. cruzi* in the southern cone of South America and the sole vector in southern Peru. It is a highly synanthropic insect found most often in poor, rural households (*3**,**4*). However, in Arequipa, a city of 850,000 inhabitants in the arid highlands of southern Peru, *T. infestans* and *T. cruzi* have become periurban and urban problems.

Since 1991, *T. infestans* has been the target of an elimination program known as the Southern Cone Initiative (*5*). Member countries of this initiative have controlled or eliminated transmission of Chagas disease by spraying households with pyrethroid insecticides (*6**–**9*). In 2002, the Arequipa Regional Ministry of Health began a spray-based vector control program after an infant in a periurban community died from acute Chagas disease. This initiative, in contrast to those in other parts of the southern cone, is concentrated in and around the city rather than in rural areas. Novel measures may be necessary to prevent vector reinfestation after insecticide application in densely populated environments.

Urbanization of *T. infestans* has been observed elsewhere in South America (*10**–**12*), and other Chagas disease vectors have been observed in cities (*13**,**14*). Nevertheless, little is known about the epidemiology of Chagas disease transmission in and around cities. To tailor vector control strategies for the urban setting, we conducted a study to identify determinants of triatomine infestation and population density in a periurban community of Arequipa. We also examined triatomines for *T. cruzi* to gain a better understanding of the spatial distribution of potential Chagas disease transmission in the community.

## Methods

### Study Area and Population

**Figure 1:**
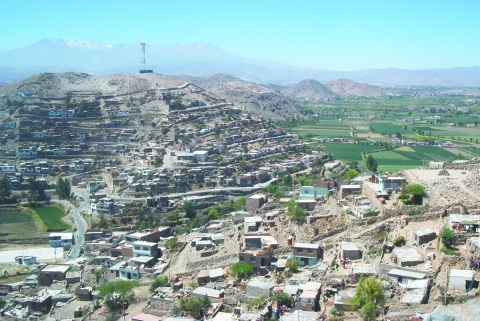
High density of homes in the periurban community of Guadalupe, Arequipa, Peru, November 2004.

Arequipa is located at an elevation of 2,300 m at the foot of an active volcano (16.44° S, 71.59° W). The area is arid; rain is scarce and falls almost exclusively from December through February. Santa Maria de Guadalupe and Alto Guadalupe (hereafter referred to together as Guadalupe) are 2 of hundreds of communities located on hillsides on the outskirts of Arequipa (Figure 1). The communities are *pueblos jovenes*, settlements built by displaced families, many of whom migrated from rural areas to the city out of economic necessity after agrarian reform in 1969. Migrants relocated to *pueblos jovenes* in Arequipa in even greater numbers to escape terrorism from 1980 to 1995 (*15*). In preliminary analyses of survey data from 1,444 schoolchildren living in Guadalupe and surrounding communities, 71 (4.9%) had serologic evidence of *T. cruzi* infection (N. Bowman, pers. comm.). The community of Guadalupe consists of 397 dwellings that house ≈2,550 people in an area of 14.1 ha (2,800 households/km^2^). Typical households consist of a human dwelling (bedrooms, kitchens, living rooms, and storage rooms) plus an enclosed yard. Roofs of the human dwellings are fully stuccoed or of corrugated metal. Walls consist of a wide variety of materials including *sillar*, a white, porous rock of compounded volcanic ash. Most yards share stone walls with neighboring households, though some back directly up against the basalt (a volcanic stone) rubble of the steep hillside. Neither community underwent systematic insecticide application before this study.

### Study Design

The entomologic survey was conducted in coordination with the first round of household insecticide application by the Arequipa Ministry of Health Vector Control Program, from November 15 to December 8, 2004. Ministry of Health personnel sprayed each house and all peridomestic structures with deltamethrin powder suspended in water at a rate of 25 mg/m^2^ (K-othrine, Bayer, Lima, Peru). After insecticide application, 2 trained triatomine collectors systematically searched each room of the human dwelling, animal enclosure, and remaining peridomestic area for a total of 1 person-hour. Because pilot studies showed marked variation in vector infestation and density within dwellings, data were collected at the level of individual rooms and animal enclosures. An adult from each household responded to a structured questionnaire regarding insecticide usage, cleaning practices, and potential triatomine hosts in each room of the dwelling and each animal enclosure. A collector recorded all construction materials used for each site. Household position was determined with a handheld global positioning system unit with an accuracy of 10 m (Garmin Corporation, Olathe, KS, USA). The protocol was reviewed by the Centers for Disease Control and Prevention's institutional review board.

Triatomines captured from each site were stored separately on ice packs until processing at the National University of San Agustin. Vectors were counted by site, stage, and sex (for adults). Live and moribund fifth instar and adult triatomines were examined for *T. cruzi* consecutively for each site until 1 positive insect was found, 10 negative insects had been examined, or all available insects had been examined, whichever came first. The sampling scheme was designed to detect *T. cruzi* in each site of collection with 80% power if >20% of insects were infected. We followed the procedures for examining triatomines for *T. cruzi* outlined in Gürtler et al. (*16*). Briefly, intestinal contents of the insects were extracted by applying pressure to the lower abdomen of the triatomine with forceps. Extracted material was then diluted in 1 drop of saline solution and examined under a microscope at 400× magnification.

### Data Analysis

Two outcome variables were examined: *T. infestans* presence (a binary outcome) and *T. infestans* population density as estimated by the number of insects captured in 1 person-hour (a continuous count outcome). Each outcome was examined separately for rooms in human dwellings and animal enclosures. In univariate analyses, associations between triatomine presence and independent variables were evaluated with the χ^2^ test for binary variables and Kruskal-Wallis trend test for ordinal and continuous variables. All variables with p value <0.20 in univariate analyses, as well as other likely confounders, were considered in multivariate analyses (*17*). Multivariate models were fit with generalized estimating equations (GEEs). A spatial variogram was used to guide selection of a correlation structure for the GEE analysis (*18*). In the absence of correlation among observations from adjacent households, an exchangeable correlation structure was assumed to adjust confidence intervals for repeated observations from the same household. Nonsignificant variables were dropped sequentially from the multivariate models on the basis of their Wald scores. Analyses were performed in Stata version 8 (StataCorp LP, College Station, TX, USA) and R version 2.1 (http://www.r-project.org).

Analyses of estimated triatomine density were limited to infested rooms and animal enclosures. To compare the mean number of vectors captured during each timed search, we used zero-truncated negative binomial regression, a method appropriate for analyses of count data in which observations of zero are excluded (*19*). Because the data were overdispersed, the zero-truncated negative binomial distribution fit the data better than the zero-truncated Poisson distribution based on the likelihood-ratio test. GEE methods for zero-truncated negative binomial regression are not available. Therefore, for households with >1 infested room or animal enclosure, 1 site only, selected at random, was included in each analysis to maintain independence of observations. All variables with p<0.20 in univariate analyses and other likely confounders were considered for inclusion in a multivariate models of the same type. Analyses were performed in Stata version 8.0.

The spatial K function of Ripley was used to test for spatial clustering of infested households (*20*). Conceptually, the K function measures the expected number of households within a set distance of any given household. The difference between the K function that summarized spatial distribution of *T. infestans*–positive households and the K function that summarized the distribution of *T. infestans*–negative households was calculated. A difference in K functions of greater than zero suggests spatial clustering of positive households (*21*). The analysis was repeated at 30 spatial scales from 10 to 300 m, and for each spatial scale, 99% tolerance limits around the observed difference in K functions for positive and negative households were determined through simulation (*21**,**22*). Clustering of households with *T. cruzi*–positive *T. infestans* was assessed in the same manner. A weighted version of the spatial K function was used to test for clustering of the number of triatomines caught in each house (*23*).

## Results

**Figure 2:**
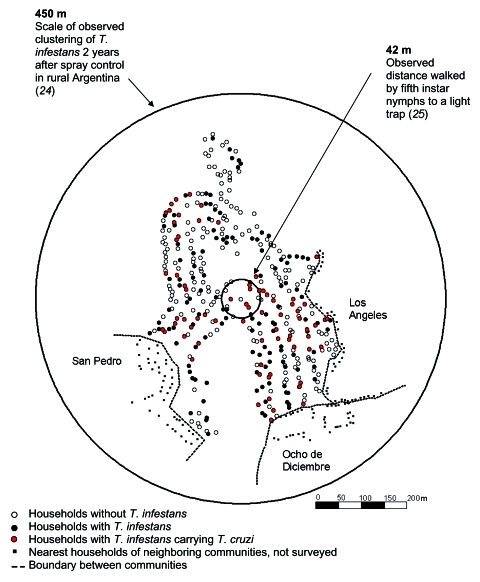
Map of households with Triatoma infestans and Trypanosmona cruzi–infected T. infestans in Guadalupe, a periurban community of Arequipa, Peru. Concentric circles are drawn around a house near the center of Guadalupe and represent parameters of T. infestans dispersal observed in rural areas (*24**,**25*). The nearest houses of neighboring communities are included for reference.

Of 397 households in Guadalupe, 374 (94.2%) were sprayed and surveyed, and 194 (52.0%) were infested with triatomines (Figure 2). Seventy-two households (19.3%) harbored triatomines infected with *T. cruzi.* Triatomines and *T. cruzi*–infected triatomines were present in human dwellings and peridomestic areas. Of 1,424 rooms in human dwellings surveyed, 241 (17%) were infested with vectors and 54 (3.8%) contained *T. cruzi*–infected triatomines. Of 803 animal enclosures, 107 (13%) were infested with vectors and 31(3.9%) had insects infected with parasites. A total of 5,398 triatomines were captured, 2,270 in human dwellings and 3,128 in peridomestic areas. The colonization indices (number of sites with triatomine nymphs/number of sites with triatomines) for rooms and animal enclosures were 76% and 93%, respectively.

At the time of the survey, 263 (70.0%) households in Guadalupe kept domestic animals. We recorded 1,700 guinea pigs, 1,295 rabbits, 819 chickens, 469 sheep, 358 dogs, 135 cats, 126 turkeys, 70 cows, and 34 pigs. Stuccoed enclosures generally housed guinea pigs (29 of 34, correlation coefficient 0.30), chicken-wire enclosures housed rabbits (54 of 134, correlation coefficient 0.18), and adobe enclosures housed sheep (15 of 39, correlation coefficient 0.17). Other types of enclosures contained a range of animals, but none predominated (all correlation coefficients <0.15). Most animals, including large animals, were kept in enclosures on the roof or in the yard of the household; only 66 households (18.7%) allowed some companion animals to sleep inside at night.

### *T. infestans* in Animal Enclosures

Several potential risk factors for triatomine infestation in animal enclosures were identified in univariate analyses (Table 1). In the multivariable logistic model, wire mesh enclosures were one fifth as likely to be positive than were enclosures of all other materials. Enclosures built wholly or partially of stacked brick or adobe were significantly more likely to be infested than other enclosures. Mortar between units of stone or brick did not significantly reduce the likelihood of infestation. Guinea pig enclosures were 1.69 times as likely to harbor vectors than were other enclosures; chicken enclosures were significantly less likely to be infested than were other enclosures. All 34 fully stuccoed enclosures were negative for *T. infestans* and were omitted from multivariate analyses.

**Table 1:** Risk factors for *Triatoma infestans* in animal enclosures in a periurban community of Arequipa, Peru*

Risk factor		% of enclosures (n = 803)	Univariate analysis		Multivariate analysis†	
			OR (95% CI)	p value	Adjusted OR (95% CI)	p value
Animal host						
	Guinea pig	24	1.54 (0.95–2.4)	0.057	1.69 (1.05–2.74)	0.031
	Rabbit	23	1.48 (0.91–2.4)	0.088	1.52 (0.93–2.49)	0.099
	Chicken	18	0.28 (0.12–0.62)	0.001	0.36 (0.16-0.80)	0.012
No. animals (mean)‡		5.5		0.29§		
Material						
	Wire mesh only	17	0.17 (0.04–0.46)	0.008	0.18 (0.06–0.53)	0.002
	Unmortared brick	19	2.96 (1.84–4.70)	0.0001	2.02 (1.23–3.29)	0.005
	Adobe	5	2.73 (1.19–5.88)	0.005	2.52 (1.18–5.39)	0.017
	Mortared brick, *sillar*, or basalt	12	0.50 (0.19–1.11)	0.08	0.50 (0.21–1.17)	0.11
	Unmortared *sillar*‡	30	1.69 (1.08–2.62)	0.14		
	Stucco¶	4	0.00 (0.00–0.70)	0.02		
Insecticide use‡		15	1.09 (0.59–1.85)	0.77		

*OR, odds ratio; CI, confidence interval. †Models were fit with generalized estimating equations appropriate for repeated measures in households. ‡Dropped from multivariate model. §Kruskal-Wallis trend test for continuous variables. ¶Fully stuccoed enclosures were omitted from the multivariate model because none were positive for *T. infestans*.

Analyses of triatomine population density included 76 infested enclosures, each from a different household. The average number of insects caught per enclosure was 21.9. Enclosures with guinea pigs had an average of 33.9 insects; wire mesh enclosures averaged 6.7 insects (Table 2). In the multivariate zero-truncated negative binomial model, the presence of guinea pigs was associated with a 2.4-fold increase in estimated triatomine density, and wire mesh enclosures were estimated to harbor only 9% as many triatomines as enclosures of any other material. Some materials in which insect collection was difficult showed lower vector densities.

**Table 2:** Determinants of population density of *Triatoma infestans* in animal enclosures in infested households of a periurban community of Arequipa, Peru*

Risk factor		Mean no. triatomines captured (range)	Univariate zero-truncated negative binomial regression		Multivariate zero-truncated negative binomial regression	
			Ratio (95% CI)	p value	Adjusted ratio (95% CI)	p value
Animal host						
	Guinea pigs	33.9 (1–343)	2.25 (0.9–5.8)	0.09	2.38 (1.0–5.7)	0.05
	No animals present	2.6 (1–7)	0.08 (0.02–0.32)	<0.01	0.15 (0.04–0.57)	<0.01
	Chickens†	28.2 (1–79)	1.38 (0.23–8.4)	0.70		
	Turkeys†‡	6.0 (1–15)	0.18 (0.02–1.5)	0.11		
No. animals†§			1.0 (0.92–1.22)	0.65		
Material						
	Wire mesh only	6.7 (1–10)	0.21 (0.02–1.75)	0.15	0.09 (0.01–0.6)	0.02
	Unmortared basalt	11.8 (1–109)	0.38 (0.15–0.96)	0.04	0.35 (0.15–0.80)	0.01
	Mortared brick, basalt, and *sillar* only	6.5 (3–15)	0.20 (0.03–1.3)	0.09	0.21 (0.04–1.1)	0.06
	Unmortared brick†	23.3 (1–90)	1.13 (0.46–2.8)	0.80		
	Adobe†	21.0 (1–112)	0.94 (0.22–4.0)	0.94		
Insecticide use†		29.8 (1–112)	1.1 (0.45–2.7)	0.80		

*Triatomine density was estimated by timed capture of insects by trained collectors. OR, odds ratio; CI, confidence interval. †Dropped from the multivariate model. ‡1 turkey enclosure, which contained 598 *T. infestans*, was considered an outlier and left out of analysis. §No mean is given for continuous variables.

### *T. infestans* in Rooms of the Human Dwelling

In the multivariate logistic model, the relative odds of infestation in rooms of the human dwelling increased multiplicatively by 1.6 for each additional person sleeping in a room (Table 3). Rooms in which an animal slept were nearly twice as likely as rooms without animals to be positive. Likelihood of being infested was less than one third for fully stuccoed rooms, 1.6 times greater for rooms built with mortared *sillar,* and 1.8 times greater for those built with mortared brick. An infested guinea pig enclosure and yard were both associated with infestation inside the human dwelling.

**Table 3:** Risk factors for *Triatoma infestans* in rooms of human dwellings in a periurban community of Arequipa, Peru*

Risk factor		% of rooms (N = 1,424)	Univariate analysis†		Multivariate logistic regression†	
			OR (95% CI)	p value	Adjusted OR (95% CI)	p value
Host						
	No. persons sleeping in room			<0.001‡	1.63 (1.48–1.79)	<0.001
	Animal sleeping in room	5.1	2.79	<0.001	1.90 (1.10–3.28)	0.021
Peridomestic infestation						
	Guinea pig enclosure	9.1	1.70 (1.07–2.63)	0.014	2.23 (1.30–3.82)	0.004
	Yard	11.7	1.41 (0.92–2.12)	0.09	2.10 (1.27–3.46)	0.004
	Sheep enclosure§	5.3	2.29 (1.31–3.90)	0.001		
Material						
	Mortared brick	19.0	2.39 (1.73–3.30)	<0.001	1.76 (1.15–2.71)	0.01
	Mortared *sillar*	20.2	2.00 (1.44–2.76)	<0.001	1.60 (1.04–2.47)	0.033
	Fully stuccoed	41.7	0.25 (0.17–0.38)	<0.001	0.27 (0.17–0.44)	<0.001
	Unmortared *sillar*§	26.5	1.21 (0.81–1.78)	0.32		
Insecticide use§		43.8	0.91 (0.68–1.22)	0.53		

*OR, odds ratio; CI, confidence interval. †Models were fit with generalized estimating equations appropriate for repeated measures in households. ‡Kruskal-Wallis trend test for continuous variable. §Dropped from the multivariate model.

A random selection of 156 infested rooms, each from a different household, was included in the analysis of vector density (Table 4). Rooms had significantly lower estimated vector densities than did animal enclosures (Kruskal-Wallis p = 0.0001); the overall average number of insects collected in rooms was 8.9. In the multivariate model, the number of insects captured increased by 42% for each person sleeping in the room. Rooms in which animals, mainly dogs and cats, slept had an estimated 5.2 times as many insects as rooms without animals. Rooms with fully stuccoed or adobe walls had significantly fewer triatomines than rooms made of other materials. The density of vectors in rooms of mortared brick, basalt, or *sillar* was not significantly different from that in other rooms after controlling for covariates.

**Table 4:** Determinants of population density of *Triatoma infestans* in rooms of human dwellings in a periurban community of Arequipa, Peru*

Risk factor		Mean no. triatomines captured (range)	Univariate zero-truncated negative binomial regression		Multivariate zero-truncated negative binomial regression	
			Ratio (95% CI)	p value	Adjusted ratio (95% CI)	p value
Host						
	Each person sleeping in room†		1.58 (1.29–1.93)	<0.001	1.42 (1.16–1.72)	0.001
	Animal sleeping in room	22.12 (1–124)	7.34 (2.20–24.54)	0.001	5.23 (1.56–17.47)	0.007
Material						
	Fully stuccoed walls and ceiling	3.03 (1–20)	0.065 (0.03–0.16)	<0.001	0.11 (0.04–0.31)	<0.001
	Adobe	1.73 (1–4)	0.055 (0.013–0.24)	<0.001	0.15 (0.032–0.73)	0.019
	Mortared brick, *sillar*, or basalt	12.62 (1–124)	2.25 (1.08–4.71)	0.03	0.80 (0.35–1.84)	0.599‡
	Unmortared brick, *sillar*, or basalt§	11.85 (1–103)	1.58 (0.65–3.87)	0.313		
Insecticide use§		9.74 (1–124)	1.20 (0.32–2.44)	0.808		

*Triatomine density was estimated by timed-capture of insects by trained collectors. CI, confidence interval. †No mean is given for continuous variables. ‡The variable describing mortared brick, *sillar*, and basalt was left in the final model because it represents a possible intervention and was therefore a principal variable of interest. Removing the variable does not greatly affect the estimates for the other parameters or the significance level of those estimates. §Dropped from the multivariate model.

### Spatial Analysis

**Figure 3:**
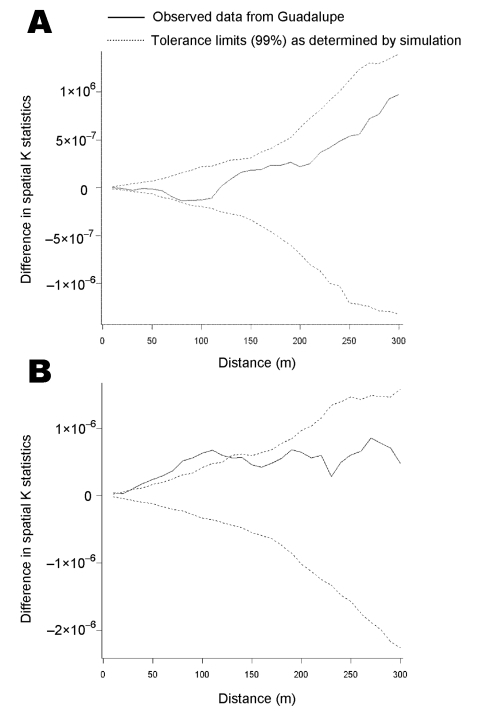
Clustering analysis of A) houses with Triatoma infestans and B) houses with Trypanosoma cruzi–infected T. infestans in a periurban community of Arequipa, Peru.

Triatomine-infested households were not significantly clustered at any of the spatial scales examined (Figure 3A). We saw no evidence of clustering in the estimated population density of triatomines across the study site. Households with triatomines infected with *T. cruzi*, however, were significantly more clustered than households without infected insects. The difference in K functions exceeded 99% tolerance limits at all but 1 spatial scale (20 m) from 10 to 140 m (Figure 3B).

## Discussion

Elimination of *T. infestans* from Arequipa may be impeded by the ease with which the vector can return to sprayed households in the densely populated urban environment. Just 2 years after blanket deltamethrin spraying in a rural community in Argentina, *T. infestans* was found in sites clustered within 450 m of a putative source population (*24*). If the range of *T. infestans* redispersion is similar in Guadalupe, a single residual population of vectors would put every household in the community, as well as many households in 3 neighboring communities, at risk for vector reinfestation. *T. infestans* nymphs can walk >42 m (*25*) and can easily climb across or through crevices in the stone walls separating the densely packed houses of Guadalupe (2,800 households/km^2^). In the rural Argentina site, with 42 households/km^2^ (*26*), vector redispersion was thought to be through flight of adult insects (*24*). *T. infestans* adults usually fly only under specific conditions (*25*). In densely populated urban and periurban sites, walking is likely to be the principal mode of redispersion, and reinfestation is likely to be much more rapid than in rural settings.

Vector reinfestation typically begins in the peridomestic environment, where domestic animals are kept (*27*). The guinea pig, a staple source of protein in the Andes for thousands of years (*28*), and reportedly a reservoir of Chagas disease in Peru (*29**–**31*), is the most numerous domestic animal in Guadalupe. As our data demonstrate, guinea pig presence is also a determinant of peridomestic *T. infestans* infestation. Enclosures where guinea pigs were housed were more likely to be infested and, when infested, harbored twice as many vectors as other enclosures. Schofield hypothesizes that *T. infestans* population growth slows when host protective behavior, such as scratching and swatting, limit the insects' ability to complete a blood meal (*32**,**33*). Incompletely fed triatomines have delayed molting (*34*) and are more likely to migrate (*25**,**35*); vector population sizes thereby decrease without an increase in insect deaths (*33*). Compared with other animals, guinea pigs may be less able to repel feeding vectors. Their habit of pressing against enclosure walls may also facilitate triatomine feeding and increase vector population growth.

Chickens are associated with infestation and increased triatomine density in rural settings, where they typically range and roost freely (*4**,**36*). Cecere et al. suggest that confining chickens might reduce triatomine populations (*36*); in Guadalupe, space constraints force most households to keep chickens enclosed. The result seems to be a decrease in the importance of chickens as hosts for *T. infestans*. Chickens hunt triatomines by sight (*34*) and may be more able to detect and catch insects from the walls of urban enclosures than from roosting materials in rural settings.

The materials used to build animal enclosures were stronger predictors of *T. infestans* presence than were the type of animals housed in the enclosure. Many materials cheaply or freely available in Arequipa, such as unmortared brick, *sillar*, and basalt, provide ample refuge for insects. Adobe was less common but was also associated with an increased risk for infestation. Fully stuccoed enclosures were never infested in Guadalupe but are costly to build. Replacing small animal enclosures of brick, *sillar*, basalt, and adobe with inexpensive wire mesh structures, which are refractory to triatomine colonization, may be the most feasible intervention to slow or prevent vector reinfestation.

The presence and density of triatomines in rooms of human dwellings are critical determinants of the risk for Chagas disease transmission to humans (*4**,**36**,**37*). In Guadalupe, the number of persons who slept in a room was the principal predictor of infestation and a determinant of vector population density. Simple interventions to decrease domestic triatomine populations in rural areas, such as keeping animals outside at night (*38*) and improving roofing materials (*39*), will likely have limited effect in Guadalupe. Although the presence of companion animals in rooms at night was associated with a 5-fold increase in vector density, animals were allowed to sleep inside in only 5% of rooms. Nearly all roofs in the community were of corrugated metal or other materials that do not provide refuge for triatomines. The only housing intervention that is likely to have a substantial effect against domiciliary triatomines in Guadalupe is completely stuccoing rooms. Schofield and Marsden showed that completely stuccoing a house could eliminate *T. infestans* from the human dwelling within 3 years (*40*). In Guadalupe, stuccoed rooms were only a fourth as likely to be infested and harbored a tenth the population of vectors compared with rooms of other materials. However, stuccoing must be complete to be effective; rooms in which mortar was used to fill the gaps between brick, *sillar*, and basalt were significantly more likely to be infested with the vector than rooms of other types.

Our study had several limitations. Triatomine collection was dependent on the excito-repellant effect of deltamethrin spray. In some materials, especially the unmortared basalt of the hillside, insecticide did not reach all refuges of triatomines, and our vector collections may have been incomplete. *T. infestans* can survive for many months without feeding, and insect population density may be more influenced by past, rather than present, host populations (*4*). We did not have information to evaluate the effect of previous animal populations on size of *T. infestans* populations at the time of spraying. Identification of households with triatomines infected with *T. cruzi* was limited by the number of insects captured at each site of collection, and the number of insects examined varied between households. The power of the analyses of estimated vector densities was diminished because we considered only 1 enclosure and room per household to maintain independence of observations.

Spatial analysis suggests that while the vector is distributed across Guadalupe, Chagas disease transmission is likely to be clustered. Households with *T. cruzi*–infected triatomines showed significant clustering. Many aspects of the complicated periurban ecology of reservoir, vector, and parasite populations could lead to spatial clustering of *T. cruzi* without clustering of its vector, but the most parsimonious explanation is a basic difference in the speed of vector and parasite dispersion. Guadalupe is a young community; 81% of households were constructed in the past 20 years. While triatomines may have had sufficient time to infest and colonize most suitable habitats in Guadalupe and may be considered endemic, *T. cruzi* may still be spreading from 1 or multiple points of introduction in a more epidemic fashion.

Many communities similar to Guadalupe are awaiting insecticide application. Acute cases of Chagas disease have been reported from communities in different parts of the city (Arequipa Regional Ministry of Health, unpub. data), though transmission of *T. cruzi* in these areas is likely focal. Timely, coordinated insecticide application is imperative to control Chagas disease in southern Peru and must be accompanied by effective surveillance for vector reinfestation. Improvement of peridomestic small animal enclosures with materials refractory to triatomine infestation could greatly increase the likelihood of eliminating the vector from the city.

## References

[1] World Health Organization. The World Health Report 2003, annex 2. Deaths by cause, sex and mortality stratum in WHO regions, estimates for 2002. 2003 [cited 2005 Nov 21]. Available from http://www.who.int/whr/2003/en/Annex2-en.pdf

[2] Kirchhoff LV, Weiss LM, Wittner M, Tanowitz HB. Parasitic diseases of the heart. Front Biosci. 2004;9:706–23. 10.2741/1255 14766402

[3] Zeledon R, Rabinovich JE. Chagas' disease: an ecological appraisal with special emphasis on its insect vectors. Annu Rev Entomol. 1981;26:101–33. 10.1146/annurev.en.26.010181.000533 6791582

[4] Cohen JE, Gürtler RE. Modeling household transmission of American trypanosomiasis. Science. 2001;293:694–8. 10.1126/science.1060638 11474111

[5] Dias JC, Silveira AC, Schofield CJ. The impact of Chagas disease control in Latin America: a review. Mem Inst Oswaldo Cruz. 2002;97:603–12. 10.1590/S0074-02762002000500002 12219120

[6] Schofield CJ, Dias JC. The Southern Cone Initiative against Chagas disease. Adv Parasitol. 1999;42:1–27. 10.1016/S0065-308X(08)60147-5 10050271

[7] Lorca M, Garcia A, Contreras MC, Schenone H, Rojas A. Evaluation of a *Triatoma infestans* elimination program by the decrease of *Trypanosoma cruzi* infection frequency in children younger than 10 years, Chile, 1991–1998. Am J Trop Med Hyg. 2001;65:861–4. 11791988 10.4269/ajtmh.2001.65.861

[8] Chagas disease. Elimination of transmission. Wkly Epidemiol Rec. 1994;69:38–40. 8155515

[9] Silveira A, Vinhaes M. Elimination of vector-borne transmission of Chagas disease. Mem Inst Oswaldo Cruz. 1999;94(Suppl 1):405–11. 10.1590/S0074-02761999000700080 10677766

[10] Gajate P, Pietrokovsky S, Abramo Orrego L, Perez O, Monte A, Belmonte J, *Triatoma infestans* in greater Buenos Aires, Argentina. Mem Inst Oswaldo Cruz. 2001;96:473–7. 10.1590/S0074-02762001000400006 11391418

[11] Albarracin-Veizaga H, de Carvalho ME, Nascimento EM, Rodrigues VL, Casanova C, Barata JM. Chagas disease in an area of recent occupation in Cochabamba, Bolivia. Rev Saude Publica. 1999;33:230–6. 10.1590/S0034-89101999000300003 10456995

[12] Vallve SL, Rojo H, Wisnivesky-Colli C. Urban ecology of *Triatoma infestans* in San Juan, Argentina. Mem Inst Oswaldo Cruz. 1996;91:405–8. 10.1590/S0074-02761996000400003 9070399

[13] Zeledon R, Calvo N, Montenegro VM, Lorosa ES, Arevalo C. A survey on *Triatoma dimidiata* in an urban area of the province of Heredia, Costa Rica. Mem Inst Oswaldo Cruz. 2005;100:507–12. 10.1590/S0074-02762005000600002 16302059

[14] Ramsey JM, Alvear AL, Ordonez R, Munoz G, Garcia A, Lopez R, Risk factors associated with house infestation by the Chagas disease vector *Triatoma pallidipennis* in Cuernavaca metropolitan area, Mexico. Med Vet Entomol. 2005;19:219–28. 10.1111/j.0269-283X.2005.00563.x 15958028

[15] Pease GYF. Breve historia contemporánea del Perú. 2nd ed. México: Fondo de Cultura Económica; 1999.

[16] Gürtler RE, Cohen JE, Cecere MC, Lauricella MA, Chuit R, Segura EL. Influence of humans and domestic animals on the household prevalence of *Trypanosoma cruzi* in *Triatoma infestans* populations in northwest Argentina. Am J Trop Med Hyg. 1998;58:748–58. 9660458 10.4269/ajtmh.1998.58.748

[17] Hosmer DW, Lemeshow S. Applied logistic regression. 2nd ed. New York: Wiley; 2000.

[18] Thomson MC, Connor SJ, D'Alessandro U, Rowlingson B, Diggle P, Cresswell M, Predicting malaria infection in Gambian children from satellite data and bed net use surveys: the importance of spatial correlation in the interpretation of results. Am J Trop Med Hyg. 1999;61:2–8. 10432046 10.4269/ajtmh.1999.61.2

[19] Lee AH, Wang K, Yau KK, Somerford PJ. Truncated negative binomial mixed regression modelling of ischaemic stroke hospitalizations. Stat Med. 2003;22:1129–39. 10.1002/sim.1419 12652558

[20] Ripley B. The second-order analysis of stationary point processes. J Appl Probab. 1976;13:255–66. 10.2307/3212829

[21] Diggle PJ, Chetwynd AG. Second-order analysis of spatial clustering for inhomogeneous populations. Biometrics. 1991;47:1155–63. 10.2307/2532668 1742435

[22] Waller LA, Gotway CA. Applied spatial statistics for public health data. Hoboken (NJ): John Wiley & Sons; 2004.

[23] Getis A. Interaction modeling using second-order analysis. Environment and Planning. 1984;A16:173–83. 10.1068/a160173

[24] Cecere MC, Vazquez-Prokopec GM, Gürtler RE, Kitron U. Spatio-temporal analysis of reinfestation by *Triatoma infestans* (Hemiptera: Reduviidae) following insecticide spraying in a rural community in northwestern Argentina. Am J Trop Med Hyg. 2004;71:803–10. 15642975 PMC1351234

[25] Vazquez-Prokopec GM, Ceballos LA, Kitron U, Gürtler RE. Active dispersal of natural populations of *Triatoma infestans* (Hemiptera: Reduviidae) in rural northwestern Argentina. J Med Entomol. 2004;41:614–21. 10.1603/0022-2585-41.4.614 15311452 PMC1351236

[26] Vazquez-Prokopec GM, Cecere MC, Canale DM, Gürtler RE, Kitron U. Spatiotemporal patterns of reinfestation by *Triatoma guasayana* (Hemiptera: Reduviidae) in a rural community of northwestern Argentina. J Med Entomol. 2005;42:571–81. 10.1603/0022-2585(2005)042[0571:SPORBT]2.0.CO;2 16119545 PMC1382187

[27] Cecere MC, Gürtler RE, Canale D, Chuit R, Cohen JE. The role of the peridomiciliary area in the elimination of *Triatoma infestans* from rural Argentine communities. Rev Panam Salud Publica. 1997;1:273–9. 10.1590/S1020-49891997000400003 9149523

[28] Salinas M. Crianza y comercialización de cuyes. Lima (Peru): Ediciones Ripalme; 2002.

[29] Herrer A. Importancia del cobayo como reservoriode la enfermedad de Chagas en la región sudoccidental. Rev Med Exp. 1955;9:45–55.

[30] Acosta HM, Ferreira CS, de Carvalho ME. Human infection with *Trypanosoma cruzi* in Nasca, Peru: a seroepidemiological survey. Rev Inst Med Trop Sao Paulo. 1997;39:107–12. 10.1590/S0036-46651997000200008 9394524

[31] Cordova E. Investigation of Chagas disease in Peru. Epidemiological study in the Tambo. valley (Matalaque district. Department of Moquegua). I. Preliminary observations. 1958–1959. Bol Chil Parasitol. 1961;16:54–9. 13695517

[32] Schofield CJ. Density regulation of domestic populations of *Triatoma infestans* in Brazil. Trans R Soc Trop Med Hyg. 1980;74:761–9. 10.1016/0035-9203(80)90196-0 7010697

[33] Schofield CJ. The role of blood intake in density regulation of populations of *Triatoma infestans* (Klug) (Hemiptera: Reduviidae). Bull Entomol Res. 1982;72:617–29. 10.1017/S0007485300008646

[34] Schofield CJ. Nutritional status of domestic populations of *Triatoma infestans.* Trans R Soc Trop Med Hyg. 1980;74:770–8. 10.1016/0035-9203(80)90197-2 7010698

[35] Lehane MJ, Schofield CJ. Field experiments of dispersive flight by *Triatoma infestans.* Trans R Soc Trop Med Hyg. 1981;75:399–400. 10.1016/0035-9203(81)90103-6 7034312

[36] Cecere MC, Gürtler RE, Chuit R, Cohen JE. Effects of chickens on the prevalence of infestation and population density of *Triatoma infestans* in rural houses of north-west Argentina. Med Vet Entomol. 1997;11:383–8. 10.1111/j.1365-2915.1997.tb00426.x 9430119

[37] Rabinovich JE, Wisnivesky-Colli C, Solarz ND, Gürtler RE. Probability of transmission of Chagas disease by *Triatoma infestans* (Hemiptera: Reduviidae) in an endemic area of Santiago del Estero, Argentina. Bull World Health Organ. 1990;68:737–46. 2127382 PMC2393169

[38] Gürtler RE, Chuit R, Cecere MC, Castanera MB, Cohen JE, Segura EL. Household prevalence of seropositivity for *Trypanosoma cruzi* in three rural villages in northwest Argentina: environmental, demographic, and entomologic associations. Am J Trop Med Hyg. 1998;59:741–9. 9840591 10.4269/ajtmh.1998.59.741

[39] Cecere MC, Gürtler RE, Chuit R, Cohen JE. Factors limiting the domestic density of *Triatoma infestans* in north-west Argentina: a longitudinal study. Bull World Health Organ. 1998;76:373–84. 9803588 PMC2305757

[40] Schofield CJ, Marsden PD. The effect of wall plaster on a domestic population of *Triatoma infestans.* Bull Pan Am Health Organ. 1982;16:356–60. 6819871

